# Implementation evaluation of a teledermatology virtual clinic at an academic medical center

**DOI:** 10.1186/s43058-023-00508-1

**Published:** 2023-10-27

**Authors:** Meenal K. Kheterpal, Ethan D. Borre, Udeyvir Cheema, Matilda W. Nicholas, Edward W. Cooner, Donna Phinney, Kelly Gagnon, Leah L. Zullig, Heather A. King, Elizabeth J. Malcolm, Suephy C. Chen

**Affiliations:** 1 Department of Dermatology, Duke University Medical Center, DUMC Box 3135, Durham, NC 27710 USA; 2 Department of Population Health Sciences, Duke University School of Medicine, Durham, NC USA; 3 Duke University School of Medicine, Durham, NC USA; 4 Duke Primary Care, Duke University School of Medicine, Durham, NC USA; 5 Duke Telehealth Office, Duke University Health System, Durham, NC USA; 6 Performance Services, Duke University Health System, Durham, NC USA; 7 Center of Innovation to Accelerate Discovery and Practice Transformation, Durham VA Health Care System, Durham, NC USA; 8 Division of General Internal Medicine, Department of Medicine, Duke University School of Medicine, Durham, NC USA

**Keywords:** Teledermatology, Implementation science, Barriers and facilitators

## Abstract

**Background:**

Teledermatology (TD) is an evidence-based practice that may increase access to dermatologic care. We sought to use the Exploration, Preparation, Implementation, and Sustainment (EPIS) and the Reach, Efficacy, Adoption, Implementation, and Maintenance (RE-AIM) frameworks to evaluate implementation of TD at Duke.

**Methods:**

The EPIS and RE-AIM frameworks were deployed to design and implement a TD program that leveraged the strengths of the Duke University Health System and addressed previously reported barriers to implementation of store-and-forward and synchronous TD models. In the resultant hybrid TD model, trained primary care providers (PCPs) sent e-comm referrals with clinical and dermatoscopic images to dermatology. These e-consults were reviewed asynchronously and patients were scheduled for a synchronous video visit with dermatology within days. Dermatologists managed the patient plan. This hybrid TD model was piloted at four primary care clinics. Pertinent outcomes from a TD-adapted RE-AIM framework were tracked using electronic health record data. Patient satisfaction was assessed using a post-video visit survey (*n* = 18). Implementation barriers and facilitators were also collected through provider surveys (*n* = 24 PCPs, *n* = 10 dermatologists, *n* = 10 dermatology residents).

**Results:**

At four PCP clinics throughout 9/1/2021–4/30/2022, there were 218 TD referrals. Video visits occurred on average 7.5 ± 0.5 days after referral and 18/18 patients completing the post-visit survey were satisfied. Adoption varied between clinics, with one placing 22% of all dermatology referrals as TD and another placing 2%. The primary PCP barriers to TD were time burdens, lack of fit in clinic flow, and discomfort with image taking. Top-endorsed potential facilitating interventions included allowing for rash referrals without dermoscopy and assurance for clinical evaluation within 3 days.

**Conclusions:**

The use of implementation science frameworks allowed for identification of system and contextual strengths which informed the hybrid TD pilot. Barriers and facilitating interventions will provide guidance for expansion and ongoing maintenance of TD.

**Supplementary Information:**

The online version contains supplementary material available at 10.1186/s43058-023-00508-1.

Contributions to the literature

- Teledermatology is widely recognized as an evidence-based practice to expand access to high-quality dermatologic care, but its optimal implementation in an academic medical center remains unknown
- Few published studies demonstrate the application of an implementation framework to measure and improve teledermatology implementation.
- We provide among the first studies to systematically investigate implementation outcomes of a hybrid teledermatology process and map primary care provider barriers and facilitators.
- Our outcomes framework may be used by other centers implementing teledermatology and the identified barriers and facilitators may be prioritized to optimize implementation success.

## Introduction

Limited patient access to dermatologic care remains a problem across many regions in the USA, and teledermatology (TD) has shown promise in reducing substantial patient wait times while achieving similar patient outcomes and satisfaction to in-person care [1–7]. Two common formats of TD include (1) store-and-forward TD in which a remote dermatologist reviews patient images at a separate time from the patient visit and forwards their clinical recommendation to the referring provider; and (2) synchronous TD in which dermatologists review the patient chart and conduct a video visit with the patient to confirm their diagnostic suspicion and directly counsel the patient [8]. While there has historically been more research around store-and-forward TD, recent Medicare payment changes allowing for reimbursement parity for video and in-person visits due to COVID-19 have popularized the synchronous TD video visit format [2, 9–11].

Furthermore, while the effectiveness of TD has been previously demonstrated, the barriers and facilitators to its implementation are less well understood [12–14]. While, to our knowledge, there have not been randomized control trials investigating the clinical effects of TD, to the extent that increased access to effective dermatological care improves health and quality of life TD implementation would be an effective intervention. There are numerous complex factors related to synchronous TD’s successful implementation and understanding of optimal implementation conditions will allow for better dissemination of the evidence-based practice [14–16]. Previously identified barriers in the implementation of synchronous TD include video quality and other technological difficulties, while facilitators include direct physician–patient communication [8, 14]. Conversely, store-and-forward TD faces barriers of limited reimbursement and decreased physician–patient communication, while benefitting from scheduling convenience for providers and a potential for superior image quality [8, 14]. Factors that have been related to positive implementation of any TD model have included integration with medical records and providing for adequate follow-up [13].

It is clear that TD solutions are not one-size-fits-all. Health systems and other clinical entities would be well served by evaluating their capabilities in an organized and structured manner prior to committing resources for the deployment of a TD system. Implementation frameworks can provide the desired systemic approach; two of which are the Exploration, Preparation, Implementation, and Sustainment (EPIS) and Reach, Effectiveness, Adoption, Implementation, and Maintenance (RE-AIM) frameworks. EPIS involves identifying outer system (e.g. government policies, payer reimbursement) and inner context factors (e.g. technological capabilities, provider sentiment) to plan each of the four implementation phases [17, 18]. The exploration phase considers the health needs to be addressed and evidence-based practices to target them. The preparation phase involves developing a plan and conducting outreach to understand implementation considerations and the implementation and sustainment phases include the initiation of the intervention and its continued use. RE-AIM, on the other hand, is an evaluative framework that has been used extensively in the translation of medical literature into everyday practice [13]. It involves identifying and measuring outcomes related to each of its five domains to determine the impact of an intervention. However, while previous TD publications have addressed specific factors of RE-AIM, few have evaluated a TD intervention across multiple or all domains [13].

**Fig. 1:**
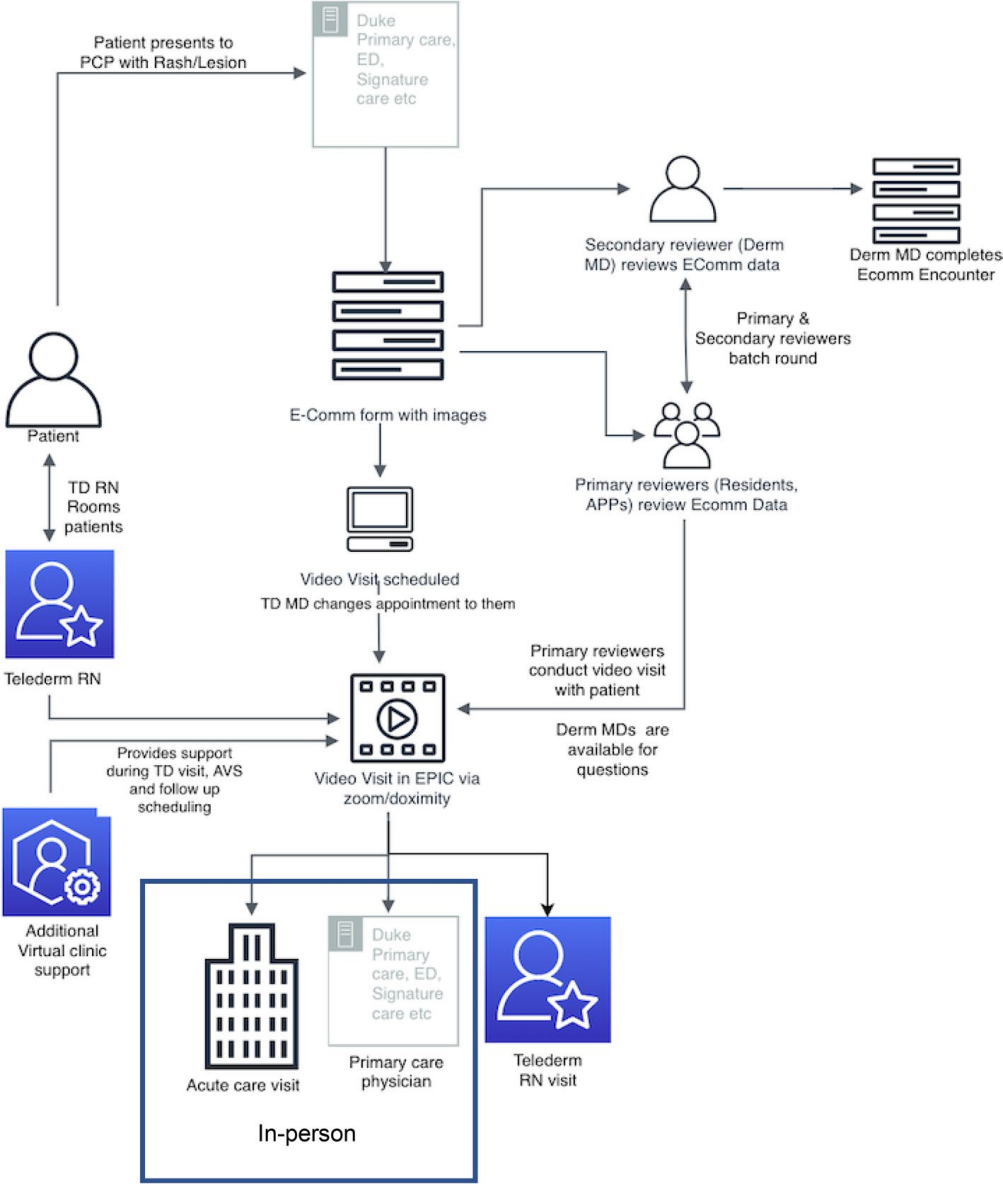
Teledermatology virtual clinic process depiction. Figure 1 outlines the hybrid teledermatology virtual clinic process. The process begins with the patient (labeled figure on left) presenting to their primary care provider (PCP) with a rash or lesion at a Duke Primary Care site. The PCP then completed an e-consult encounter form with images and the Duke Telehealth office contacts the patient to schedule a video visit with the dermatology team within 3–5 days. Prior to the video visit, the dermatology team (an attending dermatologist with a resident or advanced practice provider) reviews the e-consult form with associated dermoscopy and clinical images. Before the visit, patient is reminded of the visit and an option to e-check in is sent through patient portal. On the day of the visit, the TD nurse virtually initiates the video call, rooms the patient in the virtual waiting room, reviews intake questionnaire, and notifies the dermatology team to initiate visit. The primary dermatology team then conducts the video visit with the patient and recommends treatment. Options for follow-up include PCP follow-up for low-risk visits, TD nurse follow up for moderate complexity visits or in-person follow-up for complex visits or for concerning lesions and rashes requiring biopsy. The telephone-conducted TD nurse follow-up can vary from 2 to 6 weeks, dictated by the TD attending and based on the expected treatment response rate. If treatment is unsuccessful, the TD nurse has the option to schedule a follow-up TD visit versus an in-person visit for the patient. The blue box indicates the in-person portion of teledermatology. APP: advanced practice provider; AVS: after visit summary; E-comm: e-consult; ED: emergency department; Derm MD: attending dermatology; PCP: primary care provider; RN: nurse; Signature care: Duke concierge medicine service; TD: teledermatology

These frameworks were deployed by Duke Dermatology to address patient wait times of > 6 months (for both primary care referrals and new patients) through implementation of a hybrid TD virtual clinic in four Duke Primary Care (DPC) pilot sites beginning in September, 2021. In the hybrid care model, primary care providers (PCPs) were trained to take clinical and dermatoscopic images (Fig. 1) which were incorporated into an e-consult to dermatology. The e-consult was reviewed by the TD team and a synchronous video visit with the patient was subsequently scheduled to occur at least 3 days after the e-consult. This model was conceived to combine the superior image quality and greater convenience for providers of the store-and-forward model with the direct patient interaction and reimbursement parity of the synchronous TD model [8].

In this study, we sought to leverage the EPIS and RE-AIM frameworks to design and assess the preliminary implementation of the TD virtual clinic as well as map barriers and potential facilitators to guide its expansion and future maintenance. Our findings may provide a guide for other centers considering implementing hybrid TD video visits.

## Methods

We used a quality-improvement study design and followed the Standards for Quality Improvement Reporting Excellence [19]. All surveys were distributed via email and administered through Qualtrics; data were stored on a secure server.

### Setting and participants

Duke Dermatology and Duke Primary Care implemented the TD virtual clinic in 4 pilot primary care clinics from September 2021 through April 2022. All clinics were in located Durham, NC, had between 8 and 16 full-time PCPs, and conducted between 14,000 and 28,000 patient encounters during the study period (Table 1). These primary care clinics were selected from 32 Duke Primary Care clinics for their prior successful implementation of e-comm referrals for other specialties at Duke; these clinics were also similar with regard to strong buy-in from their medical directors. Participants in this study included dermatologists and PCPs that participated in the TD clinic.

**Table 1:** Pilot Site Encounter Information 9/1/2021–4/30/2022

Variables	Clinics			
	**A**	**B**	**C**	**D**
Clinic setting^a^	Urban	Urban	Urban	Urban
Full-time PCPs at clinic, n	8	16	15	8
Total encounters during study period, *n*	14,839	27,436	22,951	16,390
Encounters by gender, *n* (%)				
Female	9579 (65%)	16,851 (61%)	13,181 (57%)	8797 (54%)
Male	5260 (35%)	10,585 (39%)	9770 (43%)	7593 (46%)
Encounters by race, *n* (%)				
White	10,767 (73%)	15,675 (57%)	15,790 (69%)	12,246 (75%)
Black	2845 (19%)	9537 (35%)	6686 (29%)	1982 (12%)
Other	1227 (8%)	2224 (8%)	475 (2%)	2162 (13%)
Total dermatology referrals during study period, *n*	259	490	422	283

*Abbreviation*: *PCP* primary care provider

^a^Urban and rural definitions based on 2010 census data and clinic county

### Intervention

#### Development of intervention

Leadership across Duke Dermatology, Duke Health, and Duke Primary Care collaborated to define the TD virtual clinic process (Fig. 1). Implementation planning was undertaken using the Exploration, Preparation, Implementation, Sustainment (EPIS) framework [17, 18, 20]. The exploration phase began with a goal to address the Duke Dermatology new patient and referral wait time of more than 6 months. Outer contexts such as reimbursement parity for synchronous video visits and availability of grant funding along with inner context factors of strong support for TD from leadership at Duke Health and within the Duke Dermatology department paved the way for a pilot hybrid TD program. In the preparation phase, available resources were evaluated to determine readiness. Outer contexts considered at this stage included information about previous TD implementations collected via literature review and informal communications with leadership at other health organizations. These informed the initial iteration of the hybrid TD clinic that was to be adopted. Inner context factors including a centralized Duke Telemedicine scheduling and support center with experience in e-consult implementation in other specialties, EHR and data aggregation abilities afforded by Duke Performance Services, and identification of physician champions further refined the model and suggested a good organizational fit. An important characteristic of EPIS is its iterative nature. As such, a TD-adapted RE-AIM framework suggested by Peracca et al. was selected to evaluate pilot implementation; additional identification of barriers and facilitators of the pilot program were identified via survey to drive sustainability of the model under EPIS [13].

#### Hybrid TD model

The hybrid TD model begins with a patient presenting to their PCP with a rash or lesion (Fig. 1). PCPs were trained to take clinical and dermatoscopic images which were combined with basic patient information before being forwarded to the TD team as an e-consult. The TD team consisted of an initial reviewer (dermatology resident or advanced practice provider, APP) who pre-reviewed the images and charts and then “batch rounded” with a TD attending. Subsequently, the initial reviewer conducted the video visit with the patient; the attending was also on the video when the initial reviewer was a dermatologist resident, but not when they were an APP. During this pilot phase, only dermatology residents served as initial reviewers; APPs will be included in the TD process with future expansion. Only adult patients were eligible for hybrid TD.

Video visits were facilitated via the Duke Telehealth office which contacted patients to schedule the visit with the dermatology team within 3–5 days. Patient reminders were also sent through the patient portal prior to the visit with an option to electronically check in. On the day of the visit, a TD nurse initiates the video call, rooms the patient in a virtual waiting room, reviews the intake questionnaire, and notifies the TD team to initiate the visit. Visits were concluded with recommendations for treatment and follow-up. Possible options for follow-up included PCP follow-up for low-risk visits, TD nurse follow-up for moderate complexity visits, or in-person dermatology follow-up for complex visits or for concerning lesions and rashes requiring biopsy.

Initial training for PCPs consisted of an introductory clinical meeting (20 min), followed by an optional learning module (20 min) to be completed by providers virtually. The training included the description of the process and specialized image capture training, in particular: types of images (forest, close-up, dermoscopy), use of complementary body parts for rashes, common pitfalls, and examples of excellent and poor images, followed by an image quiz to assess knowledge gaps. Images were taken with an iPad and compatible dermatoscope and uploaded directly to the electronic health record for transmission to the dermatology reviewing team. Notably, PCP training did not include recommendations or requirements for patients that could be referred to TD. All e-consults required clinical and dermatoscopic images. Training for TD providers was limited to operation training on the completion of e-comm encounters. Dermatologists were able to bill video visits to payers.

### Measurement and analysis

#### Implementation framework identification and use

We used electronic health record (EHR) data to measure implementation success consistent with the Reach, Effectiveness, Adoption, Implementation, and Maintenance (RE-AIM) framework [13, 21]. RE-AIM is an evaluation framework [21–23]. To prioritize implementation outcome collection of the new TD process across the RE-AIM framework, we distributed a survey of previously published potential RE-AIM outcomes to TD leadership and asked them to select the ones they saw as most relevant to the Duke context (see Table 2 for a list of implementation outcomes; four medical directors of pilot sites and three Duke Dermatology leaders) [13, 21].

**Table 2:** Previously published teledermatology RE-AIM-based implementation outcomes, adapted from Peracca et al. 2019 [13]

RE-AIM domain	Domain definition	Outcomes assessed in the current study
Patient and Provider Reach^a^	Degree to which patients and providers are impacted	**- Number of teledermatology patients by various characteristics**
		**- Percent of dermatology encounters**
		**- Number of completed teledermatology consults**
		**- Number of teledermatology consults**
		**- Number of providers trained**
Effectiveness and Process Measures	Ability of program to change patient-centric outcomes with quality of care	- Improvements in patient health outcomes
		- Diagnostic and management concordance
		**- Consult/appointment completion times/wait times and no-shows**
		- Dermatologic skill level of PCPs
		- Quality of life
		- Costs
		**- Patient/provider satisfaction**
Adoption	Degree to which program is used by end-users	- Stages of Implementation Completion
		- Understanding link between institutional readiness for change and adoption
		**- Percent of PCPs and dermatologists using teledermatology**
		- Extent to which clinics are implementing a program by understanding administrative landscape, staffing, and training needs
Implementation	Degree to which program is implemented as planned	**- Determination of detailed barriers to and facilitators of implementation**
		- Understanding link between individual and institutional readiness for change and successful implementation
		- Whether the teledermatology process is aligned with guidelines
		**- Assess different stakeholder perspectives**
Maintenance	Can program be sustained over time?	- Examination of program implementation over time including assessment of long-term funding, collaboration and commitment between leadership, staff, and the community
		- Assessment of program responsivity such as addressing workflow and access to technology
		- Assess program results (e.g., change in number of teledermatology consults/encounters over time)
		- Identification of training programs to ensure staff involvement and integration, and to address staff attrition

Outcomes in bold were considered by our study

^a^While some of these outcomes may be considered Adoption outcomes, we chose to retain the original classifications in the framework published by Peracca et al

The results of the survey identified specific outcome measures that would be evaluated for the duration of the pilot (bolded outcomes in Table 2). Pertinent reach outcomes included the number of PCPs trained, the number of initiated and completed TD consults, and the percentage of dermatology referrals submitted via e-consult. Additionally, to ensure equity in access to this hybrid TD model, the racial composition of the TD patient population would be determined and compared to the general dermatology population served at Duke. Measures of effectiveness included wait times to see a dermatology provider in the TD model, video appointment completion times, and the no-show rate for video visits. Furthermore, patient satisfaction was also considered and measured via a post-visit survey. The primary outcome to assess adoption was the percentage of PCPs using the TD model.

Implementation under the RE-AIM framework is considered together with the implementation and sustainability phases of EPIS, with the primary outcome being the identification of barriers and facilitators of the intervention from multiple perspectives. We assessed barriers and facilitators of the virtual clinic implementation using surveys distributed to PCPs and dermatology attendings and residents.

#### Measuring implementation outcomes

We created a TD dashboard to collect real-time implementation outcomes important to the leadership, prioritizing outcomes rated highly by primary care and dermatology leadership (Table 2). The dashboard aggregated TD implementation and patient outcomes from the EHR. Patient satisfaction was measured as a post-video single question that asked whether the patient felt their clinical needs were adequately addressed (options: strongly agree, agree, disagree, strongly disagree). Final extraction and analysis of EHR data occurred after the conclusion of the pilot phase (4/30/2022).

#### Identifying barriers and facilitators to TD implementation

To identify barriers important to PCP implementation of the TD virtual, we designed a ranking survey (“PCP Survey”) of potential barriers to implementation across e-consult placement and image taking (Additional file 1). A ranking survey was chosen for this purpose so that future expansion of the TD program could efficiently allocate resources to address the most pressing concerns of providers. To identify the barrier list for this survey, the study team first identified an expanded list of all potential barriers (*n* = 17) using the EPIS framework, literature review, and dermatologist input. Since the four pilot site medical directors were also users of TD and, additionally, were able to get a direct sense for barriers faced by their clinicians, their first-hand insights were then used to select the barriers most relevant to the hybrid TD model. These barriers were included in the final “PCP Survey” (see Additional file 1 for complete survey) sent to 73 providers at the pilot sites 5 months after TD implementation began (2/11/2022). The 73 providers included full-time PCPs across all pilot sites as well as newly enrolled TD sites, regardless of whether they had submitted a TD consult or not. The responses used for analysis were limited to the pilot site PCPs based on their clinic site name and their experience with the hybrid model.

In the same “PCP Survey,” we proposed 11 potential interventions, informed by the literature, to facilitate TD virtual clinic referral for PCPs (see Additional file 1 for complete survey) [14]. For each potential facilitator, we asked the respondent to indicate whether they strongly disagreed, disagreed, agreed, or strongly agreed the intervention would facilitate implementation of TD. Upon compiling the survey results, ranking questions were scored with one point assigned to the barrier identified as most significant, two points to the second most significant, and so on. Facilitator questions were scored using a Likert scale from 1 to 4, with 1 corresponding to strongly disagree and 4 to strongly agree. The average score of each barrier and facilitator was then used to rank them and the top 4—or 5 in the event of a tie—were taken as key barriers and interventions. These key barriers to our TD process were mapped to potential implementation strategies.

We sent a “TD Provider Survey,” a version of the “PCP Survey” modified for relevance, to 10 attending dermatologists and 15 residents staffing the TD clinic to understand barriers to their implementation of the TD process. While TD attendings were asked to feedback on the same facilitators of TD used in the “PCP Survey,” dermatology residents were not asked to provide such feedback on their “TD Provider Survey” form. While APPs will be involved in the TD initial reviewing team in the future, they were not involved in TD during this pilot phase and therefore were not surveyed. The survey was sent to TD providers 7 months after TD implementation began (4/4/2022); results were analyzed using the same methodology as the “PCP Survey;” however, only the top three barriers and facilitators were selected as key measures because the modified ranking lists of the “TD Provider Survey” contained fewer overall measures.

## Results

### Patient and provider reach

During 9/1/2021–4/30/2022 at the four pilot clinics, a total of 218 e-consults were placed (Table 3; 154 lesions and 64 rashes) making up 15% of 1454 total (ambulatory in-person + TD) referrals placed by pilot sites to dermatology during the pilot phase. One hundred seventy-one video visits were completed, and the process involved 10 attending dermatologists, 15/15 resident dermatologists, and 30 primary care providers. Of the 171 completed video visits, 20 (12%) were placed by Clinic A, 77 (45%) by Clinic B, 70 (41%) by Clinic C, and 4 (2%) by Clinic D. Of patients completing a video visit, 73% self-reported White race, 18% Black race, and 9% other races. This was similar to self-reported race of ambulatory referral patients to dermatology: 72% White race, 17% Black race, and 12% other races.

**Table 3:** Implementation outcomes of a teledermatology service 9/1/2021–4/30/2022

Outcome	Value
**Reach**	
Participating clinics, *n*	4
Participating attending dermatologists, *n*	10
Participating resident dermatologists, *n*	15 (total 15 residents)
Participating primary care providers, *n*	30
E-consults placed, *n*	218
Unique patients evaluated via e-consults, *n*	216
Classified as lesion, *n*	154
Classified as rash, *n*	64
Completed TD virtual clinic visits, *n*	
Clinic A	20
Clinic B	77
Clinic C	70
Clinic D	4
**Effectiveness**	
Video visits scheduled, % of all e-consults placed	85% (186/218)
Loss-to-follow-up, % of all e-consults placed	15% (32/218)
% Patient declined or unable to be reached	12% (26/218)
% Other reason	3% (6/218)
Completed video visits, % of scheduled video visits	92% (171/186)
Video visit no-shows, % of scheduled video visits	3% (5/186)
Video visit cancellations, % of scheduled video visits	5% (10/186)
E-comm referrals scheduled in ≤ 3 days	81% (151/186)
Mean time between e-comm referral placement and video visit, days	7.5 ± 0.5
Average video visit length, minutes	10.2 ± 0.2
Conversion to telephone visit, %	1% (1/171)
Patients agree clinical goals were met, % (*n* = 18)	100% (18/18)
Patients requiring downstream completed in-person appointments, % of TD virtual clinic patients	65% (111/171)
Total downstream in-person appointments, *n*	111
**Adoption**	
PCPs at participating clinics that utilized TD, % of total providers^a^	
Clinic A	50% (4/8)
Clinic B	75% (12/16)
Clinic C	87% (13/15)
Clinic D	13% (1/8)
Percent of total dermatology referrals that utilized e-consult	
Clinic A	11% (28/259)
Clinic B	19% (94/490)
Clinic C	22% (91/422)
Clinic D	2% (5/283)

*Abbreviations*: *PCP* primary care provider, *TD* teledermatology

^a^Defined as the number of unique PCPs who placed an e-consult divided by the total number of PCPs at that clinic site

### Adoption

The percentage of all PCPs at participating pilot clinics that placed at least one TD e-consult varied between pilot clinic sites: Clinics B and C had more than 75% (12/16 and 13/15, respectively) of PCPs placing at least one referral, whereas Clinic A had 50% (4/8) and D had 13% (1/8). The percentage of total dermatology referrals (ambulatory in-person + TD) that were TD also varied between clinic sites, ranging between 2% (5/283) at Clinic D and 22% (91/422) at Clinic C. In other words, Clinic D, compared to other clinics sent the fewest TD consults.

### Effectiveness and process measures

Of all e-consults, 85% (186/218) had a scheduled video visit, with 15% (32/218) unscheduled secondary to patient declining or unable to be reached. Eighty percent (151/186) of e-consults were scheduled as video visits with the dermatology team in 3 days or less, and the mean time from e-consult to video visit was 7.5 ± 0.5 days (compared to in-person wait times of 6 months). Eight percent (15/186) of scheduled video visits were cancellations or no-shows. The average video visit length was 10.2 ± 0.2 min, and 1 video visit out of 171 (1%) was converted to a telephone visit due to patient technology difficulties. All (18/18) patients who completed the satisfaction survey (170 unique TD patients with completed video visit were surveyed, but few completed the satisfaction survey within the timeframe of this analysis) indicated that their clinical goals were met during the video visit. Sixty-five percent (111/171) of video visits required a downstream in-person appointment.

### Identified PCP barriers to TD referral

**Fig. 2:**
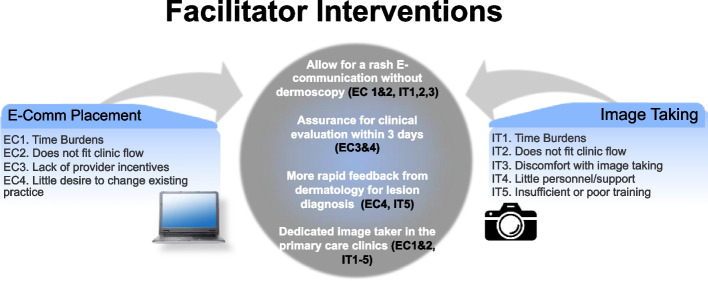
Primary care physician reported barriers to teledermatology e-consult and image taking mapped to proposed facilitating intervention. Figure 2 highlights the ranked barriers for primary care providers for both e-consult placement and image taking in the teledermatology process (left and right labeled text boxes). These barriers were ascertained through a survey. For e-consult placement, the barriers (in order of importance) were as follows: (1) time burdens, (2) does not fit in clinic flow, (3) lack of provider incentives, and (4) little desire to change existing practice. For image taking, the ranked barriers were as follows: (1) time burdens, (2) does not fit in clinic flow, (3) discomfort with image taking, (4) little personnel/support, and (5) insufficient or poor training. The central circle shows planned facilitating interventions that were mapped to alleviate barriers to e-consult placement and image taking. These facilitating interventions include (1) allowing for a rash e-consult without dermoscopy, (2) assurance for clinical evaluation within 3 days, (3) more rapid feedback from dermatology for lesion diagnosis, and (4) providing a dedicated image taker in the primary care clinics. We show which barriers these facilitating interventions map to through labels; i.e., EC1 is e-consult placement barrier 1. EC: e-consult placement; IT: image taking

Given disparate adoption rates between clinics, and that all clinics had TD referrals comprising ≤ 22% of all dermatology referrals, we assessed barriers to e-consult placement among participating PCPs through the barrier ranking survey. Twenty-four PCPs from pilot sites responded to the survey, which was 33% (24 /73) of the emailed sample. With respect to *e-consult* placement, the four highest ranked barriers were time burdens, lack of fit in clinic flow, lack of PCP incentives, and little desire to change existing practice (Fig. 2). For *image taking*, the highest ranked barriers were time burdens, lack of fit in clinic flow, discomfort with image taking, little personnel/support, and insufficient or poor image taking training. Six providers emphasized in free response that dermatoscopic and clinical image taking was the most time-consuming portion of the process, reporting difficulties retrieving the iPad from storage, logging into the EHR, and obtaining images of adequate quality.

### Identified dermatology attending and resident barriers to TD

Eight dermatology attendings responded to the “TD Provider” survey (80% response rate, 8/10). The top three endorsed barriers to TD from the dermatologist perspective were as follows: (1) video technology difficulties on the patient end, (2) concerns with ready availability of in-person follow-up slots for TD patients, and (3) concerns around compensation for TD clinic compared to in-person clinic.

Ten dermatology residents completed the survey (67% response rate, 10/15), with eight (80%, 8/10) agreeing that participating in TD is beneficial to their clinical education. The barriers ranked highest by dermatology residents were as follows: (1) concerns with ready availability of in-person follow-up slots, (2) video issues on the patient end, and (3) video issues on the provider end.

### Potential facilitators of TD referral

Of 11 proposed interventions, those most endorsed by PCPs were (1) allowing for an e-consult referral for rashes with clinical images only (no dermoscopy), while amenable to perform dermoscopy for lesions; (2) assurance that patients will receive a call to schedule the video visit within 3 days; (3) more rapid dermatologist feedback about lesion diagnosis, and (4) provision of a dedicated image taker at the primary care clinic (Fig. 2). In general, dermatology attendings agreed with these potential facilitators, ranking allowing for rash referral without dermoscopy and assurance that the patient will be contacted within 3 days among the top facilitators. We mapped the top-endorsed proposed facilitator interventions to the highest priority barriers in Fig. 2.

## Discussion

The COVID-19 pandemic forced dermatologists to implement TD quickly, with little opportunity for careful evaluation of implementation. Health care systems such as the Veterans Administration have been deploying TD for many years, but these are primarily using the store-and-forward paradigm, which continues to not be reimbursable in a scalable manner outside closed health care systems [24, 25]. A recent systematic review found no existing studies using a comprehensive implementation framework to identify factors influencing teledermatology implementation [14]. Since then, Peracca et al. recommended and subsequently deployed the RE-AIM framework to evaluate the VA consultative store-and-forward TD service to rural Veterans [13, 26]. We deployed this framework, along with EPIS, to guide and assess the implementation of a hybrid TD model at an academic health center.

Our study found that a hybrid TD virtual clinic increased patient access to dermatologic care through a reduction in wait times from 6 months to ~ 1 week, with high patient satisfaction among the 18 patients who completed the survey. Across RE-AIM, we found strong initial implementation effectiveness but with variable adoption among pilot clinic sites. In particular, Clinic D had much lower adoption measures than the other pilot sites. All pilot sites were similar with regard to qualitative measures such as the presence of physician champions and medical director support of TD. However, further investigation found that a private practice dermatology clinic was located near Clinic D. During the pilot period, this private practice had shorter wait times for in-patient new patient appointments than Duke Dermatology. Additionally, clinicians did not need to adjust their clinic flow to place a referral to this clinic, in contrast with the changes in workflow necessitated by taking images and submitting an e-comm with TD. These factors may have lead PCPs at Clinic D to refer patients to the nearby practice in lieu of TD. It is difficult to evaluate the exact number of patients or referrals placed to this outside practice with the current study approvals. Since the conclusion of the study period, wait times for new patients at the private practice have increased and adoption measures at Clinic D have been rising, although more data is necessary before attributing low adoption entirely to this phenomenon.

Even with this variable adoption, the total reach of the TD pilot program was 200 + patients and there are plans to expand to other clinics. It is interesting to note that 65% of video visits required an in-person follow-up visit. One possible explanation for this is that clinical goals were not reached during the video visit. Another explanation could be the type of consults placed to TD: 71% (154/218) of e-comms were placed for lesions. If, after a thorough review of the e-comm images and patient history during the video visit, the dermatologist determined that a biopsy of the lesion was appropriate this would necessitate an in-person follow-up. In this case, the video visit would have served the clinical purpose of determining to biopsy or not. During the pilot phase, we did not provide guidance to or place limitations on PCPs with regard to the types of chief complaints that could be referred to TD. Future work to explore differences in the percentage of downstream in-person appointments across a variety of diagnosis codes could be used to determine patients most appropriate for TD. This could also have ramifications on the financial capital required to adopt TD.

Indeed, while financial considerations and EHR integration have previously been found to be key barriers to TD adoption, the use of implementation frameworks in the early planning stages allowed for identification of factors to address these barriers directly [27]. These included strong institutional support through a centralized Duke Telemedicine scheduling and support center, partners across Duke committing financial support, the Duke Performance Services providing EHR and data aggregation support, and physician champions for operational support and clinical expertise. Importantly, the process included a “virtual clinic” model with TD nurses virtually rooming patients to reduce technology difficulties. This allowed dermatologists to evaluate and treat patients without delays. The availability of these resources may limit the generalizability of this exact hybrid TD model to other settings—particularly private practices or smaller health systems. However, the use of EPIS and RE-AIM frameworks to formally identify and address the unique contextual factors at Duke Health is one that can be applied in any setting to determine the need for, structure of, and implementation of a TD program.

Our identified barriers to implementation of a TD virtual clinic may also generalize beyond our four pilot sites. Video technology issues on the patient end, including low-quality connections, were endorsed as a barrier by dermatology attendings and residents, similar to previous reports on synchronous TD [14]. Technological improvements to telemedicine platforms and expanded access to high-speed internet may reduce this barrier in the future. Asynchronous TD addresses these technology issues as the uploading and downloading of images does not require the same level of network stability and connection as a live video feed. While our hybrid TD model used asynchronous images to address the issue of image resolution, PCPs felt uncomfortable and insufficiently supported in providing clinically useful images—particularly with dermoscopy. Other PCP barriers to the hybrid TD process—time burden and clinic fit—are commonly recognized barriers to implementation across a wide range of e-health interventions, they are particularly important in TD where dermatologists often rely on high-quality images which can require more time to provide [27]. Acceptable facilitating interventions to address all of these barriers included eliminating the dermoscopy requirement for rashes and providing a dedicated image taker. Other TD programs—hybrid or otherwise—have identified this issue through qualitative research and improved image quality to 99% acceptable images using trained, dedicated image takers (personal communication, SC Chen). Future research might take advantage of existing models of technology adoption in healthcare settings, such as the Fit between Individuals, Task, and Technology framework, to better understand barriers to technology adoption in TD [28].

We also proposed facilitating interventions and assessed their preliminary acceptability to PCPs and TD providers. As a direct result of these findings, future hybrid TD e-consults will allow for rash consultation without dermatoscopic images; this is further supported by recent studies demonstrating low perceived utility of dermatoscopic images for rashes [29]. We plan to assess this strategy’s effects on implementation outcomes and maintenance of the expanded hybrid TD program in future studies.

Our study has several limitations. First, we studied a single academic institution, so our findings may not be generalizable to other institutions in other parts of the country. We also did not address the type of hybrid TD where patients send in their own photos, without the PCP; we now offer this service but do not have enough data to analyze at this time. Second, 76% of PCPs who were administered the TD barriers survey did not respond and for confidentiality reasons we did not require them to state their clinic affiliation. However, the consistency of top-ranked barriers among all clinicians who did respond to the survey as well as site medical directors reporting their provider’s views somewhat alleviates this concern. Third, as this was a pilot phase of TD, we had relatively small sample sizes for the provider surveys. That said, the goal of this analysis is to provide a framework for evaluating early implementation of TD and facilitate responsive adaptation, which requires surveying providers early in the implementation process. In the future, increased provider participation will allow for both a greater sample size and separation of survey results by site. Fourth, we did not have socioeconomic status data on patients and did not evaluate the full patient perspective of TD, which could be done in a future study. Lastly, we did not explore maintenance outcomes given the relatively recent implementation of TD at the pilot sites but plan to continue monitoring and improving these outcomes. Since the conclusion of the pilot period, the facilitating interventions identified in this pilot analysis were implemented and the hybrid TD program has been extended to include all 32 Duke Primary Care and all 9 Duke Urgent Care centers. We hope to provide updates on the success of this widespread implementation once long-term data are available.

## Conclusions

We present a rigorous application of implementation science frameworks to evaluate TD implementation to provide a guide for other clinical settings. Overall, we found that usage of RE-AIM and EPIS frameworks allowed for the identification of internal and external factors that heavily impacted the design of the TD model and the outcomes deemed most important for evaluating its implementation. The pilot of our hybrid TD virtual clinic was highly effective at reducing patient wait times for dermatology from > 6 months to ~ 1 week; however, adoption was variable across pilot clinic sites. Barriers potentially explaining the low PCP adoption included time burdens, lack of fit in clinic flow, and difficulties with dermoscopy and clinical image taking; these will be addressed as the program expands. Dermatology practices and departments implementing a new TD program may use our implementation outcomes framework for evaluation and measurement of improvement. Additionally, programs should likely prioritize facilitating interventions to alleviate barriers identified in this study prior to implementation to optimize the success of future TD programs.

### Supplementary Information

**Additional file 1.** Primary Care Provider Survey of Barriers and Facilitators to Teledermatology.

## Data Availability

Data may be provided on request to the corresponding author.

## Abbreviations

TD: Teledermatology
DPC: Duke Primary Care
PCP: Primary Care Provider
APP: Advanced Practice Provider
EPIS: Exploration, Preparation, Implementation, Sustainment
EHR: Electronic Health Record
RE-AIM: Reach, Effectiveness, Adoption, Implementation, Maintenance
VA: Veteran’s Health Affairs
