# Supplementary material for: Implementation evaluation of a teledermatology virtual clinic at an academic medical center
Source: Implement Sci Commun. 2023 Oct 27;4:130. doi: 10.1186/s43058-023-00508-1 (PMC10612341; doi:10.1186/s43058-023-00508-1)
Supplement: Supplementary file 1 — Additional file 1. Primary Care Provider Survey of Barriers and Facilitators to Teledermatology. [file 43058_2023_508_MOESM1_ESM.docx]

**Implementation Evaluation of a Teledermatology Virtual Clinic at an Academic Medical Center**

**Supplementary Material**

Meenal K. Kheterpal, MD, MMCi

Ethan D. Borre, PhD

Matilda W. Nicholas, MD, PhD

Edward W. Cooner, MD, MBOE

Donna Phinney, MSN, RN

Kelly Gagnon, BS

Leah L. Zullig, PhD, MPH

Heather A. King, PhD

Elizabeth J. Malcolm, MD, MSHS

Suephy C. Chen, MD, MS

**Appendix 1.** Primary Care Provider Survey of Barriers and Facilitators to Teledermatology.

TD_PCP_ Barrier Facilitator Survey

This is a 17-question survey asking about your recent experience with teledermatology (virtual clinic).


The estimated time to complete this survey is less than 10 minutes.

Demographic Information

1. About how many years have you worked for Duke Primary Care?

- Less than 1 year (1)
- 1-2 years (2)
- 3-5 years (3)
- 6-10 years (4)
- 11-15 years (5)
- More than 15 years (6)

2. Please select your primary clinic affiliation among the following:

- Creedmoor (1)
- Croasdaile (2)
- South Durham (3)
- Timberlyne (4)

3. Please select your approximate use of teledermatology referrals in the past 6 months:

- Never (1)
- I have placed 1-3 referrals (2)
- I have place 3-10 referrals (3)
- I have placed greater than 10 referrals (4)

4. Over the past 6 months, approximately how many patients refuse a Telederm referral even though they have MyChart Access?

- I have not offered a Telederm referral (1)
- < 1% (4)
- 1-5% (5)
- 6-10% (6)
- > 10% (7)

4b. Optional comment: Why did your patient(s) refuse a Telederm referral?

________________________________________________________________

Barriers ranking exercise

Please rank from **most significant at the top to least significant at the bottom** the following barriers to full implementation of the placing the e-communication to dermatology referral process in your clinic.

Please drag the options to the correct position.

______ Does not fit in clinic flow

______ Little desire to change existing practice

______ Time burdens

______ Lack of awareness/understanding of E-comm process

______ Lack of provider incentives for efforts required to carry out E-comm

______ Insufficient or poor training for E-comm (6)

______ Other barriers (please write) (7)

Please rank from **most significant at the top to least significant at the bottom** the following barriers to full implementation of the dermatology image taking process in your clinic.

Please drag the options to the correct position.

______ Does not fit in clinic flow

______ Little desire to change existing practice

______ Time burdens

______ Discomfort with image taking, storage, or attaching process

______ Insufficient or poor training for image capture process

______ Little personnel/support for issues with imaging process

______ Lack of provider incentives for efforts required to carry out Imaging

______ Other barriers (please write)

Please rate how strongly you agree or disagree that the following adaptations would facilitate your use of teledermatology:

7. Changes to the e-communication referral process or image taking process that would reduce the time required of you

- Strongly disagree (4)
- Disagree (5)
- Agree (6)
- Strongly agree (7)

Optional Comment:

________________________________________________________________

8. Receiving a quarterly e-newsletter outlining clinic participation in teledermatology, in-person visits/miles saved, and % satisfactory vs. unsatisfactory images

- Strongly disagree (4)
- Disagree (5)
- Agree (6)
- Strongly agree (7)

Optional Comment:

________________________________________________________________

9. Increased messaging to patients and providers on the benefits of teledermatology to patient access

- Strongly disagree (4)
- Disagree (5)
- Agree (6)
- Strongly agree (7)

Optional Comment:

________________________________________________________________

10. Receiving more rapid feedback from the dermatologist about their lesion diagnoses

- Strongly disagree (4)
- Disagree (5)
- Agree (6)
- Strongly agree (7)

Optional Comment:

________________________________________________________________

11. Allowing for an e-communication teledermatology referral for rash without dermoscopy (clinical photographs only)

- Strongly disagree (4)
- Disagree (5)
- Agree (6)
- Strongly agree (7)

Optional Comment:

________________________________________________________________

12. Increased educational sessions on dermatologic image taking and teledermatology referral process

- Strongly disagree (4)
- Disagree (5)
- Agree (6)
- Strongly agree (7)

Optional Comment:

________________________________________________________________

13. The availability of a dedicated image-taker at your clinic for all teledermatology consults

- Strongly disagree (4)
- Disagree (5)
- Agree (6)
- Strongly agree (7)

Optional Comment:

________________________________________________________________

13. The availability of a provider at your clinic who is an expert in the teledermatology process to answer your questions

- Strongly disagree (4)
- Disagree (5)
- Agree (6)
- Strongly agree (7)

Optional Comment:

________________________________________________________________

15. The ability to provide patients with the phone number of a dedicated teledermatology navigator at the time of referral

- Strongly disagree (4)
- Disagree (5)
- Agree (6)
- Strongly agree (7)

Optional Comment:

________________________________________________________________

16. Assurance that patients will receive a call to schedule the teledermatology video visit within 3 days

- Strongly disagree (4)
- Disagree (5)
- Agree (6)
- Strongly agree (7)

Optional Comment:

________________________________________________________________

17. Financial incentive system for PCPs with high teledermatology referral rates

- Strongly disagree (4)
- Disagree (5)
- Agree (6)
- Strongly agree (7)

Optional Comment:

________________________________________________________________
